# Development and Rasch Validation of the Parental Topical Corticosteroid Phobia Scale (PTCPS) in Pediatric Eczema Care

**DOI:** 10.3390/healthcare13233160

**Published:** 2025-12-03

**Authors:** Ahmad Assiri

**Affiliations:** Department of Dermatology, Faculty of Medicine, Jazan University, Jazan 45142, Saudi Arabia; assiriah04@gmail.com

**Keywords:** topical corticosteroids, corticophobia, Rasch analysis, parental beliefs, eczema adherence

## Abstract

**Background and Objectives**: Parental fears and misconceptions about topical corticosteroids (TCS), known as TCS phobia, can hinder adherence and lead to poor eczema control in children. Despite its clinical relevance, few instruments capture this phenomenon using modern psychometric principles. This study aimed to develop and validate the Parental Topical Corticosteroid Phobia Scale (PTCPS), a brief tool grounded in Rasch measurement theory. **Methods**: A cross-sectional survey was conducted among 678 parents of children with eczema in Saudi Arabia. The five-item PTCPS was designed to reflect cognitive, emotional, and behavioral components of corticosteroid phobia. Rasch analysis using WINSTEPS assessed item fit, person and item separation and reliability, unidimensionality, and category functioning. Exploratory factor analysis (EFA) and principal component analysis (PCA) of residuals further evaluated structural validity. **Results**: All five items fit the Rasch model well (infit/outfit MnSq: 0.8–1.2), with strong item reliability (0.96) and clear item separation (4.67), indicating a well-defined item hierarchy. Person reliability was lower (0.40), suggesting limited precision in distinguishing between respondent levels. The scale showed functioning dichotomous response categories with no disordered thresholds. The eigenvalue of the first residual contrast (1.78) supported unidimensionality. Exploratory factor analysis confirmed a single-factor solution accounting for 53.0% of total variance, with substantial factor loadings (0.68–0.76) across all items, supporting structural coherence of the scale. **Conclusions**: The PTCPS is a psychometrically robust, unidimensional instrument for assessing TCS phobia in parents. Future research should validate its use across cultures, explore longitudinal stability, and assess its predictive value for treatment adherence.

## 1. Introduction

Atopic dermatitis (eczema) is one of the most prevalent chronic inflammatory skin diseases in children, affecting up to 20% of the pediatric population worldwide, with rising incidence in many countries. This high prevalence contributes to a considerable public health burden, including reduced quality of life, increased healthcare utilization, and psychosocial distress for both children and their caregiver [1,2]. Topical corticosteroids (TCS) remain the cornerstone and most accessible first-line treatment for managing eczema flares. However, despite their clinical efficacy and established safety profiles, TCS use is frequently hindered by parental concerns—collectively referred to as TCS phobia or corticophobia [3]. This phenomenon is characterized by exaggerated fears and misconceptions regarding steroid creams, including fears of skin thinning, systemic absorption, and long-term harm [4]. Globally, up to 85% of patients report some degree of corticophobia, with heightened prevalence among parents of young children. Such fears often lead to suboptimal treatment adherence and poorly controlled disease [3,4]. As a result, identifying and addressing corticophobia is critical for effective eczema care [5].

Standardized instruments have been developed to quantify this multidimensional fear. The Topical Corticosteroid Phobia (TOPICOP) questionnaire, for example, is a 12-item scale that evaluates cognitive misconceptions (“beliefs”), emotional worries (“fears”), and resultant avoidance behaviors related to TCS use [1]. Similar corticophobia questionnaires have been used internationally to gauge parental steroid concerns [1,4]. Building on this literature, a brief five-item tool can capture the core dimensions of TCS phobia with minimal respondent burden. Each item is crafted to reflect a fundamental facet of corticophobia: for instance, one item addresses visceral fear of TCS (e.g., anxiety about side effects), another targets a common misconception (e.g., “steroids are dangerous” myths), and another gauges behavioral hesitancy (e.g., reluctance to apply TCS as directed). By distilling the content domains—fear, false beliefs, and avoidance behavior—into five key questions, the instrument retains content validity while remaining succinct. A short tool is justified by its practicality: it enables quick screening of parents in busy clinics or large surveys, encouraging routine assessment of steroid fears [1]. In turn, early detection of TCS phobia allows for targeted education to dispel myths and improve adherence, ultimately bridging the gap between effective therapies and parental acceptance [3,6].

Rasch analysis represents a transformative approach in psychometric measurement, offering substantial advantages over classical test theory [7]. Its primary significance lies in the ability to convert ordinal rating scale data into interval-level measurements, enabling mathematically valid calculations such as change scores and means [8]. The model achieves measurement invariance, ensuring that person ability and item difficulty estimates remain independent of the specific sample or items used—a critical property mirroring fundamental measurement in the physical sciences [9]. Unlike traditional methods that treat all items equally, Rasch analysis accounts for unequal item difficulties and provides individualized measurement error estimates for each score level, enhancing precision in assessment [9,10]. This allows researchers to identify poorly functioning items, detect unexpected response patterns, and test instrument unidimensionality [11]. The technique has proven particularly valuable in healthcare, education, and rehabilitation settings, where accurate measurement of latent constructs such as ability, attitudes, and clinical competencies is essential [12]. By transforming qualitative responses into quantitative measurements along a continuum while maintaining methodological rigor, Rasch analysis strengthens the validity and reliability of measurement instruments [7,8,9,10,11,12].

TCS are essential in managing pediatric eczema, yet many parents exhibit TCS phobia—fear or misconceptions that compromise treatment adherence. While previous studies have described this phenomenon, few have developed psychometrically rigorous tools to measure it. Existing instruments [1,4,5,6,13,14,15,16,17,18,19] often rely on classical test theory and lack evidence of unidimensionality, item fit, and rating scale validity. Notably, no similar instruments have been developed within the context of Saudi Arabia, underscoring a critical regional gap in the literature. To address this, the current study aims to develop and validate a culturally adapted, Rasch-based instrument to assess TCS phobia in parents of children using corticosteroids. The scale was designed with specific attention to sociocultural beliefs, language clarity, and healthcare norms unique to Saudi Arabian caregivers, which may influence perceptions of TCS safety. A cross-sectional design was used, with data analyzed using WINSTEPS to evaluate fit statistics, scale functionality, and reliability. Principal component analysis and confirmatory factor analysis assessed structural validity. The anticipated outcome is a brief, valid, and reliable tool that enables precise identification of corticosteroid-related fears. This instrument will aid clinicians and researchers in addressing TCS misconceptions and improving adherence in pediatric dermatological care.

## 2. Materials and Methods

### 2.1. Study Design

This study employed a cross-sectional design to develop and validate the Parental Topical Corticosteroid Phobia Scale (PTCPS), a five-item instrument designed to assess corticosteroid-related fears among parents of children with eczema. A cross-sectional approach was selected to capture phobia perceptions at a single time point during routine clinic visits, prioritizing efficiency and feasibility over longitudinal tracking of changes in beliefs, which could be explored in future studies. The validation process was guided by Rasch measurement theory to ensure structural validity, item functioning, and reliability. This method allowed efficient access to a large, relevant population for psychometric evaluation. The study adhered to the STROBE guidelines for cross-sectional studies and followed the COSMIN standards for the development and validation of health measurement instruments.

### 2.2. Participants and Setting

Participants were parents or primary caregivers of children aged 1 to 16 years diagnosed with atopic dermatitis. They were recruited using convenience sampling from dermatology outpatient clinics located in public hospitals across five major regions in Saudi Arabia (Central, Eastern, Western, Northern, and Southern), ensuring broad geographic representation. Inclusion criteria included being the child’s legal guardian, ability to read and understand Arabic, and willingness to provide informed consent. The diverse clinical settings and national coverage enhance the generalizability of the findings. Parents of children with comorbid chronic illnesses or non-atopic dermatological conditions were excluded to reduce confounding influences on corticosteroid perceptions. Ethical approval was obtained from the institutional review boards (IRBs) of the participating hospitals, and informed consent was secured in written form prior to questionnaire administration during clinic visits. While convenience sampling facilitated recruitment, it may introduce selection bias, such as overrepresentation of parents attending clinics with more severe cases or higher health literacy. A total of 678 valid responses were collected between January 2024 and June 2025. This sample size exceeds the recommended thresholds for psychometric analyses (e.g., subject-to-item ratio of at least 10:1 for Rasch modeling [20,21] and 200–300 participants for exploratory factor analysis stability [22,23]), with a priori power calculations confirming adequacy for detecting item misfit in Rasch analysis (power > 0.80 at alpha = 0.05) [22,23].

### 2.3. Instrument Development

The Parental Topical Corticosteroid Phobia Scale (PTCPS) was developed through a multi-phase process incorporating previous scales, a targeted literature review [1,13,14,15,16,17,19,24], and expert consultation. Building on this foundation, a structured search of PubMed and PsycINFO databases using keywords such as “corticosteroid phobia,” “eczema treatment adherence,” and “parental beliefs” informed the conceptual framework [13,14,15,16,17,19,24]. Relevant items were reviewed from existing tools such as the TOPICOP questionnaire to identify content gaps and refine constructs specific to the local context, resulting in a shorter, culturally adapted scale that addresses key dimensions of TCS phobia: emotional fear, behavioral nonadherence, and misconceptions regarding harm and immune suppression. Cultural adaptation was ensured by incorporating concerns frequently expressed by Saudi parents in clinical settings, particularly around anxiety and beliefs in medication strength. An expert panel consisting of three dermatologists and two psychometricians (n = 5) independently evaluated the initial pool of items for clinical relevance, clarity, and cultural appropriateness, achieving high inter-rater agreement (Cohen’s Kappa = 0.82). Based on consensus and iterative refinement, five items were retained (see Table 1 for the final items). The items were drafted in Arabic and subjected to forward–backward translation to ensure linguistic accuracy (Table 1). A dichotomous response format (Yes = 1, No = 0) was selected to simplify administration, align with Rasch model assumptions for binary data, and facilitate clear identification of the presence or absence of phobic beliefs, though this may limit sensitivity to nuanced fear levels compared to polytomous scales. A pilot test involving 20 parents was conducted to assess item comprehension, clarity, and face validity. Feedback from this pre-test resulted in minor linguistic adjustments.

### 2.4. Data Analysis

All analyses were performed using SPSS (version 28; USA) and WINSTEPS (version 5.1; USA) [25]. Descriptive statistics were used to summarize demographic variables and item responses. Exploratory factor analysis (EFA) was conducted using principal component analysis (PCA) with varimax rotation (assuming orthogonal factors) to evaluate the underlying factor structure. The Kaiser–Meyer–Olkin (KMO) index and Bartlett’s Test of Sphericity were used to assess sampling adequacy and data suitability for factor analysis. Factors were retained based on eigenvalues > 1 and explained variance thresholds. Rasch analysis was conducted using WINSTEPS, applying the dichotomous Rasch model to examine item fit, person and item separation, reliability, and dimensionality. Infit and outfit mean square (MnSq) values between 0.6 and 1.4 were considered acceptable. Point-measure correlations (PTMA; assessing the correlation between item scores and the overall Rasch measure), item-person maps (Wright maps), and category probability curves were examined for item targeting and response category functioning. Principal component analysis of residuals was used to assess unidimensionality, with eigenvalues of the first contrast below 2.0 indicating an adequate single-factor structure [25,26]. Missing data were handled via listwise deletion, as rates were anticipated to be low (<5%) based on pilot testing. If multidimensionality was detected (e.g., eigenvalue > 2.0), items contributing to secondary dimensions would be evaluated for removal to maintain unidimensionality.

### 2.5. Ethical Considerations

All procedures followed the ethical standards of the Declaration of Helsinki. Participant confidentiality was maintained, and no identifying information was collected. Ethical approval was granted by the institutional review board of Jazan University IRB Committee (Approval Number: REC-44/07/550). Participation was voluntary, and informed consent was obtained from all respondents. Data were collected through self-administered questionnaires distributed in waiting areas of dermatology clinics.

## 3. Results

### 3.1. Characteristics

The gender distribution of the affected children was nearly equal, with 50.1% females and 49.9% males. Regarding occupation, the largest group were housewives (32.6%), followed by employees (29.6%) and teachers (23.6%). Most participants held postgraduate degrees (65.5%), while 17.6% held university degrees. The onset of eczema most occurred between 1 and 3 years of age (38.6%), followed by onset before the age of one (28.9%) (Table 2). The mean age of parents was approximately 35 years (SD = 9.63), indicating a broad age distribution among caregivers. In contrast, the mean age of children with eczema was 7.4 years (SD = 3.86), reflecting a sample concentrated in early to mid-childhood. The data highlights greater variability in parental age compared to child age, consistent with expectations for a pediatric population.

### 3.2. Clinical Characteristics and Treatment Patterns

Nearly half of the children had their eczema well controlled (47.5%), while 40.6% were partially controlled, and 11.9% were uncontrolled (Table 3). Symptom severity was reported as moderate in 47.5% of cases, mild in 37.3%, and severe in 15.2%. Regarding corticosteroid use, 47.3% of parents were previous users of topical corticosteroids (TCS), 44.0% were current users, and 8.7% had never used them. The primary source of treatment guidance was dermatologists (75.7%), followed by general practitioners (17.0%) and other sources (7.4%) (Table 3).

### 3.3. Unidimensionality (Exploratory Factor Analysis)

**Figure 1:**
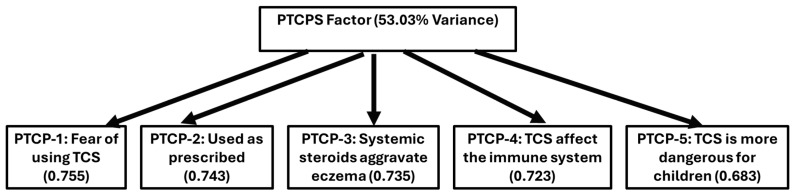
Factor structure of the Parental Topical Corticosteroid Phobia Scale (PTCPS) based on exploratory factor analysis. The single extracted factor accounted for 53.03% of the total variance and included five items (PTCP-1 to PTCP-5). Each item represents a core dimension of TCS phobia and is shown with its corresponding factor loading.

Exploratory factor analysis (EFA) was conducted on the five items of the Parental Topical Corticosteroid Phobia Scale (PTCPS) using principal component analysis (PCA). The Kaiser–Meyer–Olkin (KMO) measure of sampling adequacy was 0.758, indicating that the data were suitable for factor analysis. Bartlett’s Test of Sphericity was significant (χ^2^ = 907.05, df = 10, *p* < 0.001), confirming the appropriateness of the correlation matrix. A single component emerged with an eigenvalue greater than 1, explaining 53.03% of the total variance, supporting the unidimensional structure of the PTCPS. All five items demonstrated strong loadings on this factor (≥0.68). The highest loadings were observed for PTCP-1 (Fear of using TCS) at 0.755 and PTCP-2 (Used as prescribed) at 0.743. Communality values ranged from 0.467 to 0.570, suggesting that a moderate proportion of variance in each item was explained by the latent construct (Table 4 and Figure 1).

### 3.4. Rasch Item-Level Statistics

#### 3.4.1. Unidimensionality, Item Fit, and Rating Scale Validity

The Rasch item-level statistics demonstrated strong psychometric performance of the PTCPS. Item separation was high at 4.67, indicating excellent discrimination between items along the TCS phobia continuum and supporting a well-defined item hierarchy (Table 5). The item reliability coefficient was 0.96, suggesting that the scale is highly replicable and the item estimates are stable across samples. The mean item measure was centered at 0.00, as expected in Rasch calibration, confirming that items were appropriately scaled. Fit statistics for infit and outfit mean square residuals were 1.01 and 0.98, respectively, both falling within the acceptable range (0.7–1.3), reflecting strong conformity of items to the Rasch model. Additionally, standardized fit values (ZSTD) were within ±2, indicating no evidence of item misfit. Overall, the PTCPS items showed excellent internal consistency and alignment with Rasch model expectations.

The first residual contrast had an eigenvalue of 1.78, which accounted for 27.0% of the unexplained variance (expected: 35.5%). Since the eigenvalue is below the commonly used threshold of 2.0, it suggests that no dominant secondary dimension is present in the residuals. This supports the unidimensionality assumption of the Rasch model, indicating that the items in the PTCPS primarily measure a single underlying construct.

#### 3.4.2. The Wright Map

**Figure 2:**
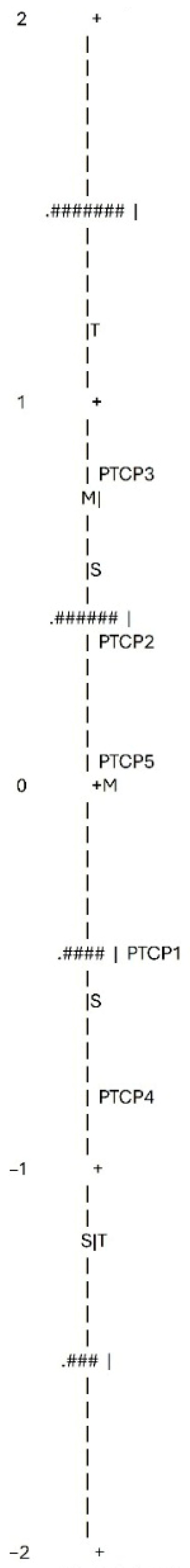
Wright Map of the PTCP Scale displaying item difficulty (right) and person ability (left) on the same logit scale. Each “#” represents 17 respondents; items range from easiest (PTCP-4) to most difficult (PTCP-3).

The Wright Map (Figure 2) provided a visual representation of the alignment between respondent TCS phobia levels and item difficulty on the PTCPS. The distribution of participants was centered around the 0 to +1 logit range, indicating moderate levels of phobia in the sample. The five items of the scale demonstrated a clear hierarchical structure, with PTCP-3 representing the most difficult item, endorsed primarily by individuals with higher phobia levels. In contrast, PTCP-4 was the easiest item, endorsed by respondents with lower levels of concern. PTCP-2 (“Used as prescribed”) and PTCP-5 occupied the middle range of difficulty, while PTCP-1 was slightly easier to endorse. Overall, the item locations matched the person distribution well, supporting the content validity and targeting of the scale. However, the clustering of respondents at higher trait levels suggests that the scale may benefit from the inclusion of additional items with higher difficulty to improve discrimination among individuals with stronger TCS-related fears.

#### 3.4.3. Category Probability Curve Results

**Figure 3:**
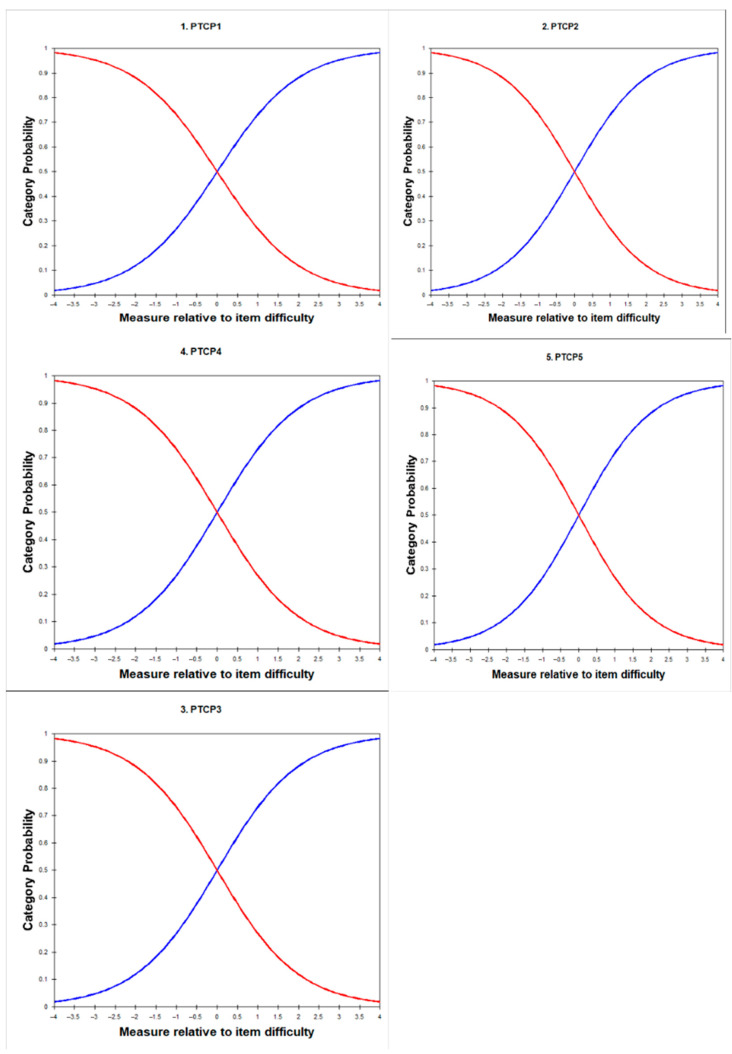
Category probability curves for the five PTCPS items. Each graph depicts the probability of selecting “Yes” (red line) or “No” (blue line) across levels of the TCS phobia trait. Curves cross near 0 logits, indicating well-functioning dichotomous categories.

The category probability curves for the five items of the Parental Topical Corticosteroid Phobia Scale (PTCPS) are presented in Figure 3. These curves illustrate the probability of endorsing each response category (“Yes” or “No”) across varying levels of the latent trait (TCS phobia), plotted relative to item difficulty in logits. For each item (PTCP-1 to PTCP-5), the curves display an orderly crossover point near 0 logits, indicating that individuals at average levels of TCS phobia are equally likely to endorse either category. The smooth, S-shaped curves for each response option reflect well-functioning dichotomous response categories. As the level of TCS phobia increases, the likelihood of choosing “Yes” rises consistently across all items. No disordered thresholds or overlapping response curves were observed, suggesting the response categories are clearly understood and appropriately utilized by respondents. This supports the validity of the scale’s binary format and its ability to capture meaningful differences in the latent construct.

#### 3.4.4. Item Category Functioning and Fit Statistics

The response category statistics for the five items of the PTCPS are summarized in Table 6. Each item demonstrated well-distributed category use, with “Yes” responses selected more frequently (55% to 72%) than “No” responses. This distribution reflects an expected directional endorsement pattern for TCS phobia. The point-measure correlations (PTMA) for all items were positive and substantial (range: 0.69–0.75), supporting internal consistency and alignment with the underlying trait. Infit and outfit mean square (MnSq) values ranged from 0.8 to 1.2, indicating acceptable fit to the Rasch model and absence of noise or redundancy in item responses. Slight elevation in outfit MnSq for PTCP4 and PTCP5 (1.1–1.2) is within tolerable limits for dichotomous items and does not compromise overall model fit. These findings affirm the stability and discriminative capacity of all five PTCPS items across the latent trait spectrum of TCS phobia.

## 4. Discussion

The present study developed and validated the Parental Topical Corticosteroid Phobia Scale (PTCPS), a unidimensional, psychometrically sound instrument for assessing corticosteroid-related fears among parents of children with eczema. Rather than focusing on numeric indicators alone, the study highlights how the PTCPS effectively captures nuanced psychological concerns—such as anxiety, avoidance, and misconceptions—regarding TCS use in pediatric care. The observed item hierarchy reveals a spectrum of parental beliefs, from mild caution to intense fear, consistent with prior findings that link corticosteroid phobia to poor adherence and suboptimal disease management. The PTCPS not only reflects a well-structured latent trait but also demonstrates cultural relevance and conceptual clarity, especially within the Saudi Arabian context, where no similar instruments had been previously developed. This underscores the broader implication of creating contextually tailored tools to address culturally influenced treatment barriers. The scale offers clinicians and researchers a focused means of identifying and addressing phobia-driven nonadherence. Thus, the PTCPS fills a critical gap in dermatological care by offering both a practical and theoretically grounded approach to tackling TCS hesitancy.

The demographic characteristics of the study sample provide important context for interpreting levels of corticosteroid phobia among parents. A substantial majority of respondents were mothers [27], aligning with existing research that identifies mothers as the primary caregivers and healthcare decision-makers for children with eczema. Most participants held postgraduate degrees [27], which may reflect increased healthcare engagement in urban clinic settings, potentially influencing both awareness and misconceptions about topical corticosteroids. Similarly to prior studies, the gender distribution of affected children was nearly equal [28], suggesting no gender-specific bias in parental reporting. The predominance of early-onset eczema—particularly between infancy and toddlerhood—is consistent with the epidemiology of atopic dermatitis, which typically manifests during the earliest stages of life [29]. This early onset may heighten parental concern about prolonged corticosteroid use. Furthermore, the sample was drawn from multiple regions across Saudi Arabia, enhancing geographic representativeness and supporting the generalizability of findings. The broad age range among parents and the concentration of child age in early childhood further underscore the relevance of this population to pediatric dermatological care.

The results of the exploratory factor analysis (EFA) provide empirical support for the unidimensionality of the Parental Topical Corticosteroid Phobia Scale (PTCPS). The acceptable Kaiser–Meyer–Olkin (KMO) value (an acceptable level) and significant Bartlett’s Test of Sphericity confirmed the adequacy of the data for factor analysis. A single factor explaining just over half of the total variance meets commonly accepted thresholds for structural validity in psychological scales [30]. All items loaded strongly on the single factor, indicating conceptual cohesion. The highest loadings were seen in items related to emotional fear and adherence behavior, consistent with literature describing these as central features of corticosteroid phobia [1]. These findings support the theoretical coherence and construct validity of the PTCPS.

The psychometric evaluation of the PTCPS using Rasch modeling supports the scale’s structural and functional validity [31]. The Wright Map revealed adequate targeting between person ability and item difficulty. Most respondents clustered within the +0 to +1 logit range, indicating moderate TCS phobia, while the items spanned a reasonable range of difficulty [32]. PTCP-3 emerged as the most difficult item, representing more nuanced or intense phobic beliefs, whereas PTCP-4 was the easiest, reflecting more common concerns. The alignment between item distribution and person ability suggests appropriate item targeting, although future iterations might benefit from adding more challenging items to enhance measurement precision at higher phobia levels [33,34].

The category probability curves (Figure 3) demonstrated well-functioning dichotomous response categories. Each item showed an orderly crossover near 0 logits, affirming balanced thresholds and interpretability [35]. The “Yes” response was more frequently endorsed, suggesting prevalent concern or phobic tendencies in the sample, consistent with literature highlighting widespread parental corticosteroid apprehension [1].

Fit statistics (Table 5) confirmed the model’s robustness. Infit and outfit MnSq values fell within acceptable ranges (0.8–1.2), indicating minimal distortion and strong item conformity [36,37]. Point-measure correlations (0.69–0.75) were all positive and strong, supporting unidimensionality and internal consistency [26,38]. PTCP-1 and PTCP-2, addressing fear and adherence, showed high alignment with the latent trait, aligning with core themes in corticosteroid phobia literature [18,39].

These findings validate the PTCPS as a reliable, unidimensional instrument capable of differentiating varying levels of TCS-related concern. Its alignment with Rasch assumptions enhances its utility in clinical and research contexts, particularly for identifying misconceptions and tailoring educational interventions [40,41,42,43,44,45]. Additionally, the development process incorporated cultural adaptation specific to the Saudi Arabian context, ensuring the instrument reflects local parental beliefs, language, and health behaviors. This cultural relevance strengthens the tool’s practical application in regional healthcare settings and supports its role in addressing context-specific barriers to effective eczema management.

This study has several limitations. First, data were collected using a cross-sectional design, limiting causal inferences. Second, the sample was restricted to one country, which may affect generalizability. Third, self-report measures may introduce social desirability bias. These limitations highlight opportunities for future research. Future studies should employ longitudinal designs to assess temporal stability and predictive validity. Cross-cultural validation in diverse populations is essential to enhance generalizability. Additionally, incorporating clinician-reported outcomes may triangulate findings and further validate the PTCPS instrument. Finally, the relatively low person reliability observed suggests limited variability in TCS phobia among participants, indicating the need for more heterogeneous samples or expanded item pools in future validation efforts.

## 5. Conclusions

Based on rigorous Rasch analysis and exploratory factor analysis, this study confirms that the Parental Topical Corticosteroid Phobia Scale (PTCPS) is a psychometrically sound instrument for measuring TCS-related fears among parents of children with eczema. The five-item scale demonstrated unidimensionality, high item reliability, well-targeted item difficulty, and meaningful person–item alignment, affirming its structural validity and interpretability. Together, these findings indicate that TCS phobia can be reliably captured using a concise, theory-driven scale. This study addresses a critical gap in the literature by offering a Rasch-validated tool that overcomes the limitations of existing instruments rooted in classical test theory. By providing a robust measure of parental misconceptions and emotional responses to topical corticosteroids, the PTCPS enhances our capacity to evaluate treatment adherence barriers and tailor educational interventions. These results extend existing models of corticosteroid phobia and support the theoretical conceptualization of TCS-related fear as a measurable, unidimensional construct. Future research should explore the scale’s responsiveness to intervention, test–retest reliability, and cross-cultural validity to strengthen its generalizability and clinical utility. The findings also invite longitudinal investigations into how parental TCS phobia evolves over time and affects long-term eczema management. Ultimately, the PTCPS provides clinicians and researchers with a targeted, evidence-based tool to address steroid hesitancy, improving patient care and therapeutic outcomes.

## Figures and Tables

**Table 1:** Final Items of the Parental Topical Corticosteroid Phobia Scale (PTCPS).

Item Number	Item Statement (English Translation for Reference)
PTCPS1	I am afraid that topical corticosteroids will harm my child’s skin.
PTCPS2	I avoid using topical corticosteroids on my child because of potential side effects.
PTCPS3	Topical corticosteroids can weaken my child’s immune system.
PTCPS4	I feel anxious when thinking about applying topical corticosteroids to my child.
PTCPS5	I believe topical corticosteroids are too strong for my child’s eczema.

**Table 2:** Sample Characteristics (n = 678).

Variable	Category	n	%
Parent with child with eczema	Yes	678	100
Parent gender	Female	479	70.6
	Male	199	29.4
Child gender	Female	340	50.1
	Male	338	49.9
Occupation	Housewife	221	32.6
	Employee	201	29.6
	Teacher	160	23.6
	Others	56	8.3
	Doctor	40	5.9
Education level	Postgraduate	444	65.5
	University	119	17.6
	Other	115	17
Disease onset age	1–3 years	262	38.6
	<1 year	196	28.9
	3–7 years	146	21.5
	>7 years	74	10.9

**Table 3:** Clinical Characteristics and Treatment Patterns (n = 678).

Variable	Category	n	%
Disease control level	Controlled	322	47.5
	Partially controlled	275	40.6
	Uncontrolled	81	11.9
Symptom severity	Moderate	322	47.5
	Mild	253	37.3
	Severe	103	15.2
TCS experience	Previous user	321	47.3
	Current user	298	44
	Never used	59	8.7
Treatment source	Dermatologist	513	75.7
	General practitioner	115	17
	Others	50	7.4

**Table 4:** Exploratory Factor Analysis Results for the PTCPS (n = 678).

Item ID	Factor Loading	Communality
PTCP-1	0.755	0.57
PTCP-2	0.743	0.552
PTCP-3	0.735	0.541
PTCP-4	0.723	0.523
PTCP-5	0.683	0.467

Extraction method: Principal Component Analysis. One component extracted.

**Table 5:** Rasch Model Summary Statistics for the PTCPS.

Statistic	Value	Interpretation
Item Separation	4.67	Excellent item discrimination; strong evidence of item hierarchy.
Item Reliability	0.96	Very high internal consistency; item estimates are stable and replicable.
Mean Item Measure	0	Items are centered around the expected Rasch model mean (logit scale).
Infit/Outfit Mean Square	1.01/0.98	Indicates strong overall item fit to the Rasch model.
ZSTD Values	±0.1 to ±1.1	All ZSTD values are within the acceptable range (<2), indicating no item misfit.
Unexplained Variance (1st Contrast)	Eigenvalue = 1.78 (27.0%)	First contrast is below the threshold of 2.0; supports unidimensionality of the scale.

**Table 6:** Item Category Response Frequencies and Rasch Fit Statistics for the PTCPS (n = 678).

Item	Category	Frequency (%)	Mean Ability	Infit MnSq	Outfit MnSq	PTMA Corr.
PTCP1	No	213 (31%)	−1.36	1	1	−0.73
	Yes	465 (69%)	1.72	0.9	0.9	0.73
PTCP2	No	274 (40%)	−1.03	1	1	−0.75
	Yes	404 (60%)	1.95	0.9	0.8	0.75
PTCP3	No	308 (45%)	−0.84	1	1	−0.74
	Yes	370 (55%)	2.07	0.9	0.8	0.74
PTCP4	No	192 (28%)	−1.38	1.1	1.1	−0.69
	Yes	486 (72%)	1.59	1.2	1.2	0.69
PTCP5	No	250 (37%)	−1.11	1	0.9	−0.73
	Yes	428 (63%)	1.84	1.1	1.1	0.73

Legend: Infit/Outfit MnSq = Mean Square Fit Statistics; PTMA Corr. = Point-Measure Correlation.

## Data Availability

The datasets used and/or analyzed during the current study are available from the corresponding author on reasonable request.
